# Association Between Primary Care Use Prior to Cancer Diagnosis and Subsequent Cancer Mortality in the Veterans Affairs Health System

**DOI:** 10.1001/jamanetworkopen.2022.42048

**Published:** 2022-11-14

**Authors:** Edmund M. Qiao, Kripa Guram, Nikhil V. Kotha, Rohith S. Voora, Alexander S. Qian, Grace S. Ahn, Sandhya Kalavacherla, Ramona Pindus, Matthew P. Banegas, Tyler F. Stewart, Michelle L. Johnson, James D. Murphy, Brent S. Rose

**Affiliations:** 1 VA San Diego Health Care System, La Jolla, California; 2 Department of Radiation Medicine and Applied Sciences, University of California, San Diego, La Jolla; 3 Division of Hematology-Oncology, Department of Internal Medicine, University of California, San Diego, La Jolla; 4 Department of Family Medicine and Public Health, University of California, San Diego, La Jolla

## Abstract

**Importance:**

Primary care physicians (PCPs) are significant contributors of early cancer detection, yet few studies have investigated whether consistent primary care translates to improved downstream outcomes.

**Objective:**

To evaluate the association of prediagnostic primary care use with metastatic disease at diagnosis and cancer-specific mortality (CSM).

**Design, Setting, and Participants:**

This cohort study used databases with primary care and referral linkage from multiple Veterans’ Affairs centers from 2004 to 2017 and had a 68-month median follow-up. Analysis was completed between July 2021 and September 2022. Participants included veterans older than 39 years who had been diagnosed with 1 of 12 cancers. Inclusion criteria included known clinical staging, survival follow-up, cause of death, and receiving care at the Veterans Affairs health system (VA).

**Exposures:**

Prediagnostic PCP use, measured in the 5 years prior to diagnosis. PCP visits were binned into none (0 visits), some (1-4 visits), and annual (5 visits).

**Main Outcomes and Measures:**

Metastatic disease at diagnosis, cancer-specific mortality (CSM) for entire cohort and stratified by tumor subtype.

**Results:**

Among 245 425 patients representing 12 tumor subtypes, mean age was 65.8 (9.3) years, and the cohort skewed male (97.6%), and White (76.1%), with higher levels of comorbidity (58.6% with Charlson Comorbidity Index scores ≥2). Compared with no prior visit, some PCP use was associated with 26% decreased odds of metastatic disease at diagnosis (odds ratio [OR], 0.74; 95% CI, 0.71-0.76; *P* < .001) and 12% reduced risk of CSM (subdistribution hazard ratio [SHR], 0.88; 95% CI, 0.86-0.89; *P* < .001). Annual PCP use was associated with 39% decreased odds of metastatic disease (OR, 0.61; 95% CI, 0.59-0.63; *P* < .001) and 21% reduced risk of CSM (SHR, 0.79; 95% CI, 0.77-0.81; *P* < .001). Among tumor subtypes, prostate cancer had the largest effect size for prior PCP use on metastatic disease at diagnosis (OR for annual use, 0.32; 95% CI, 0.30-0.35; *P* < .001) and CSM (SHR for annual use, 0.51; 95% CI, 0.48-0.55; *P* < .001).

**Conclusions and Relevance:**

In this cohort study, increased primary care use before cancer diagnosis was associated with significant decreases in metastatic disease at diagnosis and cancer-related death, with potentially the greatest difference from annual use. PCPs play a vital role in cancer prevention, and additional resources should be allocated to assist these physicians.

## Key Points

**Question:**

Is higher primary care use prior to cancer diagnosis associated with disease severity at diagnosis and long-term cancer survival?

**Findings:**

In this cohort study of 245 425 adults in the Veterans Affairs health system, annual primary care physician (PCP) use was associated with significantly reduced (39%) odds of metastatic disease at diagnosis and a significantly reduced (21%) risk of cancer-related death. Among distinct cancer subtypes with annual PCP use, 11 of 12 subtypes had a significant reduction in metastatic disease at diagnosis and 12 of 12 had a significant reduction in cancer-related death.

**Meaning:**

These findings suggest that PCPs are vital for early cancer detection, and allocating additional resources toward primary care may reduce the nationwide cancer burden.

## Teaser

This cohort study examines the association of prediagnostic primary care use on metastatic disease at diagnosis and cancer-specific mortality using multiple Veterans’ Affairs databases.

## Introduction

Cancer is the second-leading cause of death in the United States.^1^ Detecting cancers at earlier stages facilitates curative treatment and decreases cancer mortality.^2^ Primary care physicians (PCPs) provide a significant proportion of the health care aimed at early cancer detection.^3^ Consistent primary care use likely offers greater adherence to cancer screening guidelines and efficient diagnostic testing for symptomatic tumors. These preventative screening measures aid early stage-shift and improved cancer outcomes.^4^ Moreover, earlier cancer detection often spares patients from adverse effects of aggressive treatment for higher stage disease.

The importance of consistent primary care use has been increasingly emphasized in the cancer management pathway in recent years. Beyond greater access to screening and diagnostic testing, higher PCP use offers strong patient-clinician relationships for comprehensive care including follow-up, survivorship, and end-of-life care.^3^ Despite these obvious benefits, the current literature lacks national estimates of how PCP use prior to cancer diagnosis impacts cancer outcomes. Furthermore, few studies have evaluated how the benefit of early cancer detection may differ across distinct tumor subtypes nor the number of preventative visits required to constitute consistent care.

To address these shortcomings in the current literature, we conducted a large retrospective analysis using a nationwide sample of patients from the Veterans’ Health Administration (VHA). We created cohorts of patients with cancer that represented 12 distinct solid-tumor subtypes and evaluated the association of previous primary care use on presenting with metastatic disease and for cancer-specific mortality (CSM). We evaluated these end points separately for each subtype and within a combined general cancer cohort.

## Methods

### Data Source

Waivers of consent and authorization were granted by the by the University of California institutional review board and the Research and Development Committee of the VA San Diego Healthcare System. Informed consent was waived due to retrospective nature and minimal risk. The Veterans Affairs (VA) health care system stores patient-level electronic health record information for veterans, accessible through the Veterans’ Affairs Informatics and Computing Infrastructure (VINCI). Data include clinical notes, imaging, demographics, operative, and pathology reports. VINCI includes tumor registry information gathered by registrars at VA sites issued from the American College of Surgeons.^5,6^ This study followed the Strengthening the Reporting of Observational Studies in Epidemiology (STROBE) reporting guideline.

### Study Population

The study population included patients older than 39 years who had been diagnosed with solid-tumor cancer from 2004 to 2017 with a median follow-up of 68 months. Patients without complete metastatic staging, survival follow-up, cause of death, or primary payer other than the VA were excluded (eFigure in the Supplement). These covariables were extracted: age at diagnosis, gender, ethnicity, race, year of diagnosis, marital status, cancer staging variables, employment status, Charlson comorbidity scores, mean bachelor’s education percentage, and mean income. Racial and ethnic disparities in cancer care are well-documented, and these variables were also included in our modeling. We included the Charlson comorbidity score, which uses 19 categories of comorbidity,^7^ to capture common comorbid diagnoses for each patient. Mean bachelor’s education and income were obtained by tying each patient’s location to zip code-level education and income data. Healthcare Common Procedure Coding System and Current Procedural Terminology codes identified PCP visits (eMethods in the Supplement). We defined a prediagnostic observation period as the 5 years prior to cancer diagnosis. This method has been previously described.^8,9^ PCP visits prior to cancer diagnosis were binned into none (0 visits), some (1-4 visits), and annual (5 visits).

### Statistical Analysis

**Figure 1.**
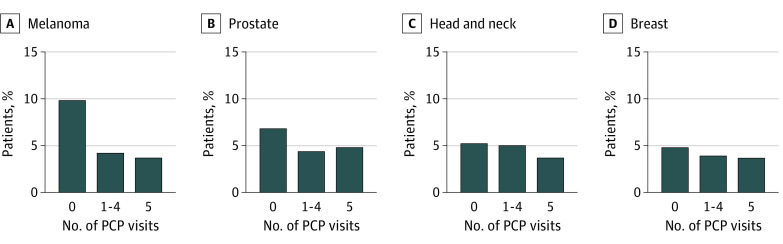
Association of Primary Care Use With Metastatic Disease at Diagnosis for Melanoma, Prostate, Head and Neck, and Breast Cancers Abbreviation: PCP indicates primary care physician.

**Figure 2.**
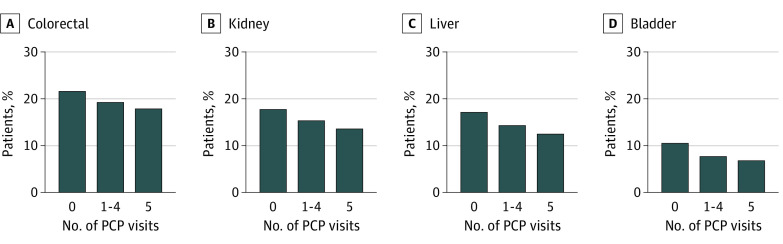
Association of Primary Care Use With Metastatic Disease at Diagnosis for Colorectal, Kidney, Liver, and Bladder Cancers Abbreviation: PCP indicates primary care physician.

**Figure 3.**
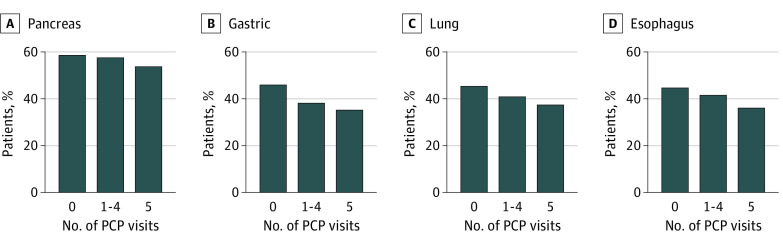
Association of Primary Care Use With Metastatic Disease at Diagnosis for Pancreas, Gastric, Lung, and Esophageal Cancers Abbreviation: PCP indicates primary care physician.

**Figure 4.**
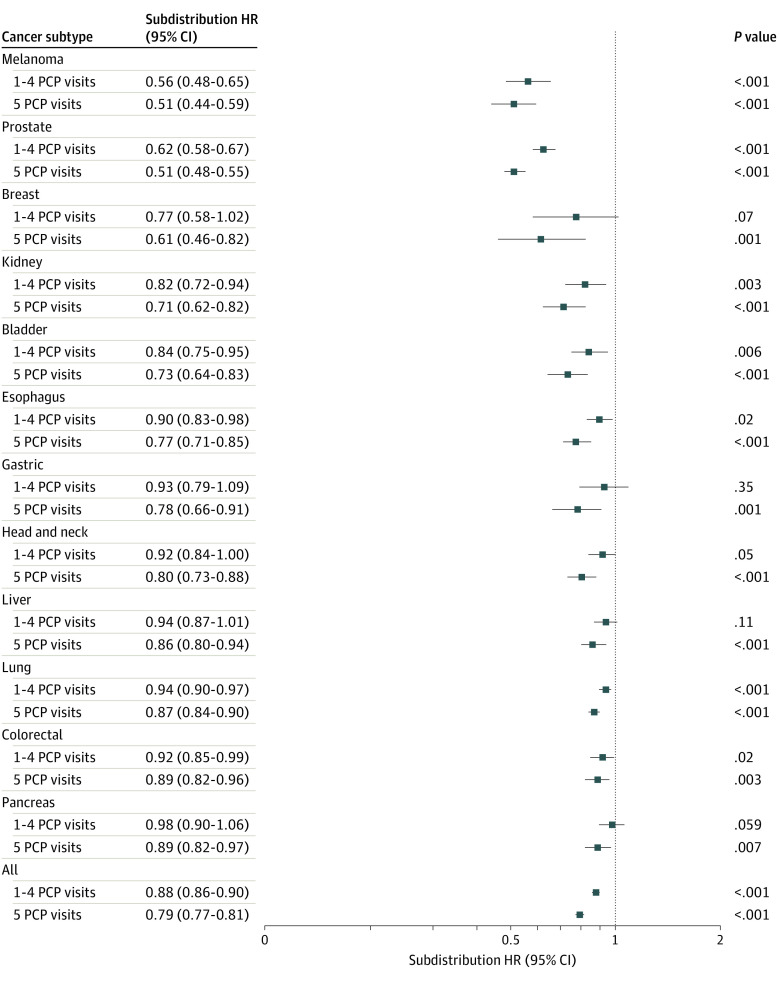
Association of Primary Care Use With Cancer-Specific Mortality Within Different Cancer Subtypes Abbreviations: HR indicates hazard ratio; PCP, primary care physician. Points represent subdistribution hazard ratio for primary care use for separate multivariable Fine-Gray competing risk regression models.

Table covariates were included in a multivariable logistic regression evaluating metastatic disease at diagnosis for the combined general cancer cohort. Tumor and nodal staging are strongly correlated with metastatic disease and were excluded from statistical modeling. The remaining Table covariates have been demonstrated as prognostic factors for cancer outcomes^10^ and were included in final modeling. Additional subcohorts were stratified by cancer subtype, and separate subtype-specific multivariable logistic regressions were performed without the cancer subtype covariate (Figure 1, Figure 2, and Figure 3). This process was repeated for CSM, using multivariable Fine-Gray competing risk regressions with noncancer death as a competing risk and CSM as the endpoint (Figure 4). A multivariable logistic regression was then performed to evaluate predictors for annual PCP use (eTable 2 in the Supplement). In our modeling, age was included as a continuous variable and all other quantitative variables were binned into categorical variables. The small proportion of missing demographic variables were grouped into the other category. All statistical tests were 2-sided, with *P* < .05 considered statistically significant. Analyses were conducted with R version 3.5.1, survival (v3.2) and ggplot2 (v3.3.3) (R Project for Statistical Computing) and completed between July 2021 and September 2022.

**Table.** Summary of Baseline Characteristics

Variable	Patients, No. (%)^a^		
	None	Some	Annual
No.	31 183	106 543	107 699
Primary care visits, count			
0	31 183 (100)	NA	NA
1	NA	16 721 (15.7)	NA
2	NA	20 396 (19.1)	NA
3	NA	22 747 (21.4)	NA
4	NA	46 679 (43.8)	NA
5	NA	NA	107 699 (100)
Age, mean (SD), y	62.3 (8.2)	64.6 (9.0)	68.0 (9.6)
Gender			
Male	30 488 (97.8)	104 129 (97.7)	104 943 (97.4)
Female	695 (2.2)	2414 (2.3)	2756 (2.6)
Race			
Black	6083 (19.5)	23 302 (21.8)	22 083 (20.5)
White	24 024 (77.0)	79 866 (75.0)	82 826 (76.9)
Other^b^	1076 (3.5)	3375 (3.2)	2790 (2.6)
Ethnicity			
Hispanic or other^c^	481 (1.5)	1734 (1.6)	1723 (1.6)
Non-Hispanic	30 702 (98.5)	104 804 (98.4)	195 970 (98.4)
Year diagnosed			
2004-2008	13 269 (42.6)	43 356 (40.7)	38 476 (35.7)
2009-2012	11 108 (35.6)	31 903 (30.0)	33 034 (30.7)
2013-2017	6787 (21.8)	31 212 (29.3)	36 189 (33.6)
Cancer subtype			
Prostate	12.908 (41.4)	42 499 (39.9)	38 049 (35.3)
Lung	4940 (15.8)	19 626 (18.4)	23 536 (21.9)
Melanoma	1322 (4.2)	3899 (3.7)	4391 (4.1)
Colorectal	4064 (13.0)	10 427 (9.8)	9328 (8.7)
Bladder	1034 (3.3)	3880 (3.6)	4712 (4.4)
Gastric	346 (1.1)	1431 (1.3)	1694 (1.6)
Kidney	1269 (4.1)	5206 (4.9)	5873 (5.5)
Esophagus	706 (2.3)	2644 (2.5)	2687 (2.5)
Head and neck	2432 (7.8)	7937 (7.4)	7190 (6.7)
Pancreas	654 (2.1)	2679 (2.5)	3197 (3.0)
Liver	1095 (3.5)	4909 (4.6)	5522 (5.1)
Breast	413 (1.3)	1406 (1.3)	1520 (1.4)
Clinical T Stage			
TX	3460 (11.1)	10 755 (10.0)	10 286 (9.6)
T1	12 485 (40.0)	47 959 (45.0)	50 267 (47.2)
T2	7697 (24.7)	25 324 (23.8)	25 264 (23.5)
T3	3794 (12.2)	11 848 (11.1)	11 658 (10.8)
T4	3747 (12.0)	10 657 (10.0)	10 224 (9.5)
Clinical N Stage			
NX	2431 (7.8)	7217 (6.8)	6481 (6.1)
N0	21 690 (69.6)	77 443 (72.7)	79 356 (73.7)
N1	2804 (9.0)	8247 (7.7)	8375 (7.8)
N2	3182 (10.2)	10 291 (9.7)	10 175 (9.4)
N3	1076 (3.5)	3345 (3.1)	3312 (3.1)
Clinical M Stage			
M0	25 121 (80.8)	88 318 (83.2)	89 374 (83.4)
M1	5966 (19.2)	17 836 (16.8)	17 777 (16.6)
Charlson			
0	11 846 (38.0)	33 261 (31.2)	24 513 (22.8)
1	3252 (10.4)	14 000 (13.1)	14 766 (13.7)
≥2	16 085 (51.6)	59 282 (55.6)	68 420 (63.5)
Employment			
Employed	6943 (22.3)	22 023 (20.7)	16 780 (15.6)
Not employed	24 128 (77.7)	84 431 (79.3)	90 896 (84.4)
Marital status			
Married	13 200 (26.9)	47 348 (28.6)	51 294 (31.3)
Single/other	35 863 (73.1)	118 162 (71.4)	112 627 (68.7)
Income, mean (SD), per $1000	50.7 (19.0)	50.1 (18.8)	50.0 (18.9)
Education, mean (SD), college degree % per zip code	15.6 (7.8)	15.6 (7.8)	15.6 (7.7)

^a^ None represents 0 previous primary care physician (PCP) visits in the 5 years prior to cancer diagnosis, some represents between 1 to 4 previous PCP visits, and annual represents 5 previous PCP visits.

^b^ Other includes American Indian, Aleutian, Eskimo, Chinese, Japanese, Filipino, Hawaiian, Korean, Vietnamese, Laotian, Hmong, Cambodian, Thai, Asian Indian, Pakistani, Micronesian, Chamorro, Guamanian, Polynesian, Tahitian, Samoan, Tongan, Melanesian, Fiji Islander, New Guinean, Other Asian, Pacific Islander, not listed, and unknown.

^c^ Other includes unknown, not listed, or Spanish surname only.

## Results

### Baseline Characteristics

Our cohort included 245 425 patients representing 12 unique tumor subtypes. Mean (SD) age at diagnosis was 65.8 (9.3) with median (IQR) follow-up of 68 (32-107) months. Prostate cancer represented the largest proportion of patients (93 456 [38.1%]), followed by lung cancer (48 102 [19.6%]) and colorectal cancer (23 819 [9.7%]). Breast cancer (3339 [1.4%]) and gastric cancer (3471 [1.4%]) had the lowest representation. In general, the cohort skewed male (239 560 [97.6%]), and White (186 716 [76.1%]), with higher levels of comorbidity (143 787 [58.6%] with Charlson Comorbidity Index scores ≥2). The Table summarizes additional characteristics.

### Primary Care Visits

Overall, previous PCP visit distribution was left-skewed, with a median (range) of 4 (0-5) previous visits. There were 107 699 patients (43.8%) who had annual PCP use and 31 183 patients (12.7%) who had no prior prediagnostic PCP visit. On multivariable analysis, older age, female gender, Black race, Hispanic ethnicity, unemployment, higher Charlson scores, and higher education were associated with statistically significant predictors for annual PCP use (eTable 2 in the Supplement).

### Metastatic Disease at Diagnosis

Among all cancer patients, 41 579 (17.0%) presented with metastatic disease. On multivariable logistic regression, older age, Black race, male, earlier year of diagnosis, no recorded Charlson score, employment, and unmarried status were associated with statistically significant higher odds of metastatic disease at diagnosis (eTable 1 in the Supplement). Based on multivariable analysis on the combined cancer cohort, some prior PCP use was associated with 26% reduced odds of metastatic disease at diagnosis when compared with no prior PCP use (odds ratio [OR], 0.74; 95% CI, 0.71-0.76; *P* < .001). Furthermore, annual visits prior to cancer diagnosis were associated with 39% reduced odds of metastatic disease at diagnosis (OR, 0.61; 95% CI, 0.59-0.63; *P* < .001).

Stratifying by tumor subtypes, higher PCP use was significantly associated with lower proportion of metastatic disease at diagnosis on unadjusted analysis (Figure 1-3). On multivariable analysis, higher PCP use was also associated with lower odds of metastatic disease. For prostate, lung, melanoma, colorectal, bladder, gastric, and kidney tumor subtypes, some PCP use was associated with a statistically significant reduction in the OR for metastatic disease at diagnosis—a total of 7 of 12 tumor subtypes. When looking at annual PCP use, all but pancreas cancer was observed to have a statistically significant reduction in the OR for metastatic disease at diagnosis (eTable 3 in the Supplement). The largest effect size for PCP use was observed for prostate cancer (OR for annual use, 0.32; 95% CI, 0.30-0.35; *P* < .001) and the lowest for pancreas cancer (OR for annual use, 0.87; 95% CI, 0.73-1.04; *P* = .12).

### Cancer-Specific Mortality

The overall cumulative incidence of CSM for the combined cohort at median follow-up of 68 (32-107) months was 28.5%. Based on multivariable survival analysis, older age, Black race, male gender, earlier year of diagnosis, Charlson score of 2 or more, unemployment, unmarried status, lower income, and lower education were associated with statistically significantly higher risk of CSM (eTable 1 in the Supplement).

Regarding prediagnostic PCP use, multivariable analysis of our combined cohort demonstrated that higher prior PCP visits were associated with a reduced risk of CSM, with a 12% reduced risk for some prior PCP use (subdistribution hazard ratio [SHR], 0.88; 95% CI, 0.86-0.89; *P* < .001) and 21% reduced risk for annual PCP use (SHR, 0.79; 95% CI, 0.77-0.81; *P* < .001).

Stratifying by tumor subtypes, higher PCP use remain statistically significant predictors for CSM in almost all tumor subtypes (Figure 4). For melanoma, prostate, kidney, bladder, esophagus, lung, and colorectal subtypes, some PCP use was associated with a statistically significant reduction in the risk of CSM—a total of 7 of 12 tumor subtypes. When looking at annual PCP use, 12 of 12 tumor subtypes were observed to have a statistically significant reduction in the SHR for CSM. The largest effect size was again observed for prostate cancer (SHR for annual use, 0.51; 95% CI, 0.48-0.55; *P* < .001), tied with melanoma (SHR for annual use, 0.51; 0.44-0.59; *P* < .001). The lowest observed effect size was for pancreas cancer (SHR for annual use, 0.89; 95% CI, 0.90-0.97; *P* = .007).

## Discussion

Primary care physicians are significant contributors toward early cancer diagnosis.^3^ Increased health care use is associated with earlier diagnosis and greater probability of curative treatment options.^2^ While the association between consistent preventative health care with improved cancer outcomes is frequently discussed among clinicians, our study represents the first—and largest—to estimate the association of prediagnostic primary health care with cancer outcomes for a variety of tumor subtypes. Our findings underscore the important relationship between consistent primary care visits and improved outcomes among patients at increased risk of cancer.

From our analysis, higher prediagnostic PCP use was associated with a significantly reduced odds for metastatic disease at diagnosis for 11 of 12 cancer subtypes and a significantly reduced risk of CSM for all 12 subtypes. When evaluating the entire cohort, patients with some prior PCP use had 26% reduced odds of metastatic disease at diagnosis and a 12% reduced risk of CSM for patients. Furthermore, annual PCP use was observed to have an even greater effect size, with 39% reduced odds in metastatic disease and 21% reduced risk of CSM. Our tumor-stratified analysis provides additional granularity for patients at increased risk of certain cancer subtypes, such as those with tumor-specific family history or environmental exposures. These cancer-specific risk estimates may help physicians provide more personalized guidance for patients at elevated risk of developing certain cancers.

Our findings reflect both the natural history of each tumor subtype as well as current diagnostic and treatment interventions. Most cancer screening originates from the primary care setting,^11,12,13^ and proper screening is associated with improved cancer outcomes.^14,15,16,17^ From our analysis, cancer subtypes that may undergo frequent screening, such as prostate, melanoma, and breast, were observed to have the greatest effect sizes for prediagnostic primary care use on our end points, whereas less screenable tumors (eg, pancreas tumor) were observed to have lower benefit. As an aside, there is debate regarding prostate-specific antigen (PSA) screening for prostate cancer, with current recommendations emphasizing shared decision-making.^18^ An explanation for our prostate cancer-specific results may be that greater adherence to annual PCP use may allow for better risk-stratification and shared-decision making for initiating PSA screening in higher risk patients. Prostate cancer screening is an evolving discussion, and the effects of PSA screening on cancer outcomes are outside the scope of the present study.

Even in cancers without well-established screening paradigms, we observed improved outcomes for patients with higher PCP use. Consistent PCP use represents an obvious and advantageous preventative measure for these occult malignancies. Interestingly, the association of PCP visits with colorectal cancer was lower than might be expected, as colorectal cancer is another highly screenable disease. One explanation may be the frequency of cancer screening performed for colorectal cancer. Depending on patient risk, colorectal screening recommendations are at 1, 3, 5, or 10-year intervals,^19^ with most opting for 5- or 10-year screening with sigmoidoscopy or colonoscopy.^20^ The benefit of PCP utilization may be obscured by the lower frequency of colorectal cancer screening, as patients with lower PCP visits may be screened just as frequently as those with higher PCP use. Nevertheless, PCPs are responsible for proper recommendation of screening, and we emphasize that for almost every tumor subtype, increasing PCP use was associated with a statistically significant reduction in presenting with metastatic disease and CSM.

Our prediagnostic period included visits between 1 to 6 years prior to cancer diagnosis. This timeframe likely captures the asymptomatic period for most of these cancers and highlights the importance of earlier workup of potential malignancies. As a patient’s primary contact with the health care system, PCPs possess a comprehensive knowledge of a patient’s lifestyle, family history, and other cancer-risk factors. This unique patient-clinician relationship allows PCPs to swiftly identify changes in patient presentation that may require a deeper malignancy workup. Furthermore, the role of the PCP in cancer prevention extends far beyond ordering diagnostic and screening tests. Higher primary care use likely both reflects and enhances patients’ overall trust in the health care system. Within our analysis, higher prediagnostic PCP use was significantly associated with improved CSM for even the most aggressive tumor subtypes. The patient-clinician trust established before cancer diagnosis likely translates to more consistent postdiagnosis follow-up, such as earlier identification of tumor recurrence. This patient-clinician relationship potentially affects patients’ decisions to pursue curative treatment with their primary oncologists as well,^21,22^ which may help explain the observed cancer-specific survival benefit in patients with higher prediagnostic PCP use.

Our analysis highlights known cancer health care disparities. Black patients are known to have poorer cancer outcomes in the general population.^23^ These patients are more likely to be uninsured and underrepresented in cancer screening and intervention trials.^24,25^ Even within an equal-access health care system such as the VHA, our combined cohort regressions showed that Black patients were at a mildly elevated risk of presenting with metastatic disease and CSM when compared with White patients (eTable 1 in the Supplement). These disparities are likely even greater in the general population, where Black patients face additional structural barriers in health care systems and reflected in underuse of cancer screening tests within this population. Improving access to preventative health care must be a priority when addressing existing, nationwide health care disparities.

Bolstering preventative visits for patients at risk for cancer remains a complex challenge. In health care systems that are already overburdened, a sharp increase in preventative medicine visits may inundate the current capacity of PCPs. Undifferentiated preventative efforts may prove costly and time-consuming to effectively implement for large populations. However, targeted cancer screening and preventative efforts have been shown to be cost-effective in numerous studies.^26^ Targeted genetic testing among high-risk breast cancer patients and mammogram screening among the general population have been shown cost-effective.^27^ Current lung cancer screening strategies and targeted screening for prostate cancer are both cost-effective measures.^28,29^ Clinician decision-making remains integral for proper identification and screening of patients at risk of developing cancer. Additional allocation of resources to support PCPs in early cancer diagnostic efforts may potentially alleviate some of the cost burdens associated with downstream cancer care.

### Limitations

This study has limitations. First, we relied on diagnostic and procedural coding to identify PCP visits and used registrar data for other variables. Misclassification of these may introduce bias into our analysis. For example, our data pipeline limited patients to first cancer diagnoses, though a small number of patients with recurrent cancer may have been misclassified. We believe this misclassification would be more likely to attenuate than heighten our regression results by reducing the effect size conveyed by PCP visits through increased randomness in our data. Second, the retrospective study design is potentially limited by unmeasured confounders, and the described association of PCP visits with our end points must be understood within this context. For instance, it is unclear whether cancer screening or prevention is discussed at each PCP visit, as time might be allocated toward chronic disease management instead. Quantifying this complex patient-clinician relationship requires additional prospective studies. In addition, patients who choose to see their PCP more often may adhere to other healthier lifestyle choices (eg, diet, exercise, etc) that decrease their cancer risk. Third, our cohort consisted of veterans, and the generalizability of this population to the general population may be limited. Additional prospective studies including balanced age and gender groups and greater minority representation are needed to validate our findings.

## Conclusions

In this cohort study of a large nationwide sample of patients, we found that increased primary care use prior to cancer diagnosis was associated with a statistically significant decrease in metastatic disease at diagnosis and CSM, with annual PCP use associated with the greatest decrease in risk. Higher PCP use was associated with significantly decreased odds of metastatic disease at diagnosis for 11 of 12 tumor subtypes and significantly decreased risk of cancer-related death in all 12 tumor subtypes. PCPs play a vital role in cancer prevention, and additional resources should be allocated to assist these physicians.

## Notes

Supplement.
eMethods.
**eFigure.** Study Cohort **eTable 1. **Full Model Outputs of Logistic Regression and Fine-Gray Competing Risk Regression for Entire Cohort **eTable 2. **Full Model Outputs of Logistic Regression for Entire Cohort and Annual Primary Care Visits as Endpoint **eTable 3.** ORs and 95% CIs for Multivariable Logistic Regressions With Metastatic Disease at Diagnosis. **eTable 4.** Comparison of Included vs. Excluded Patients
