# Supplementary material for: Association Between Primary Care Use Prior to Cancer Diagnosis and Subsequent Cancer Mortality in the Veterans Affairs Health System
Source: JAMA Netw Open. 2022 Nov 14;5(11):e2242048. doi: 10.1001/jamanetworkopen.2022.42048 (PMC9664263; doi:10.1001/jamanetworkopen.2022.42048)
Supplement: Supplement. — eMethods. eFigure. Study Cohort eTable 1. Full Model Outputs of Logistic Regression and Fine-Gray Competing Risk Regression for Entire Cohort eTable 2. Full Model Outputs of Logistic Regression for Entire Cohort and Annual Primary Care Visits as Endpoint eTable 3. ORs and 95% CIs for Multivariable Logistic Regressions With Metastatic Disease at Diagnosis. eTable 4. Comparison of Included vs. Excluded Patients [file jamanetwopen-e2242048-s001.pdf]

Supplemental Online Content

Qiao EM, Guram K, Kotha NV, et al. Association between primary care use prior to cancer diagnosis and subsequent cancer mortality in the Veterans Affairs health system. *JAMA Netw Open*. 2022:5(11):e2242048. doi:10.1001/jamanetworkopen.2022.42048

eMethods.
eFigure. Study Cohort
eTable 1. Full Model Outputs of Logistic Regression and Fine-Gray Competing Risk Regression for Entire Cohort
eTable 2. Full Model Outputs of Logistic Regression for Entire Cohort and Annual Primary Care Visits as Endpoint
eTable 3. ORs and 95% CIs for Multivariable Logistic Regressions With Metastatic Disease at Diagnosis.
eTable 4. Comparison of Included vs. Excluded Patients

This supplemental material has been provided by the authors to give readers additional information about their work.

© 2022 Qiao E et al. *JAMA Network Open.*

eMethods

For primary care utilization, Current Procedural Terminology (CPT) codes '99201', '99202', '99203', '99204', '99205', '99211', '99212', '99213', '99214', '99215', '99381', '99382', '99383', '99384', '99385', '99386', '99387', '99391', '99392', '99393', '99394', '99395', '99396', '99397', '99401', '99402', '99403', '99404', '99408', and '99409' were used.

© 2022 Qiao E et al. *JAMA Network Open.*

eFigure. Study Cohort

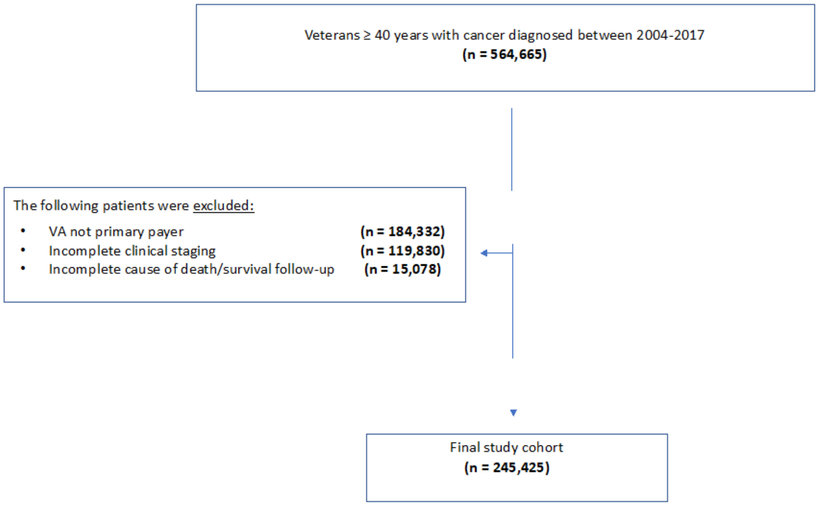

© 2022 Qiao E et al. *JAMA Network Open.*

Supplementary Table 1. Full Model Outputs of Logistic Regression and Fine-Gray Competing Risk Regression for Entire Cohort

Characteristic	Endpoint OR/SHR [95%CI]	
	Metastatic disease at diagnosis (OR)	Cancer-specific mortality (SHR)
PCP visits		
None (0 visits)	1.00	1.00
Some (1-4 visits)	0.74 [0.71-0.76]	0.88 [0.86-0.89]
Annual (5 visits)	0.61 [0.59-0.63]	0.79 [0.77-0.81]
Age at Diagnosis	1.01 [1.01-1.01]	1.02 [1.01-1.02]
Race		
White	1.00	1.00
Black	1.07 [1.04-1.10]	1.05 [1.03-1.07]
Other	1.02 [0.95-1.09]	1.01 [0.97-1.06]
Ethnicity		
Hispanic	0.95 [0.86-1.04]	0.97 [0.92-1.04]
Non-Hispanic/Other	1.00	1.00
Gender		
Male	1.00	1.00
Female	0.87 [0.80-0.95]	0.79 [0.74-0.83]
Cancer Subtype		
Prostate	1.00	1.00
Bladder	1.49 [1.37-1.61]	4.86 [4.65-5.10]
Breast	1.29 [1.09-1.53]	2.92 [2.62-3.26]
Colorectal	4.44 [4.26-4.64]	4.25 [4.09-4.40]
Esophageal	12.26 [11.55-13.01]	19.31 [18.59-20.05]
Gastric	11.50 [10.67-12.39]	11.12 [10.53-11.74]
Head and Neck	0.96 [0.89-1.04]	4.70 [4.52-4.88]
Kidney	3.39 [3.20-3.59]	3.56 [3.39-3.74]
Liver	3.20 [3.00-3.39]	14.60 [14.11-15.11]
Lung	12.64 [12.21-13.09]	18.28 [17.78-18.80]
Melanoma	1.34 [1.23-1.46]	2.63 [2.48-2.80]
Pancreas	23.89 [22.56-25.30]	33.75 [32.56-34.98]
Year of Diagnosis		
2004-2008	1.00	1.00

© 2022 Qiao E et al. *JAMA Network Open.*

2009-2012	0.98 [0.97-0.96]	0.77 [0.66-1.24]
2013-2017	0.84 [0.82-0.87]	0.46 [0.35-0.65]
Charlson Score		
0	1.00	1.00
1	0.91 [0.89-0.95]	0.97 [0.95-0.996]
2+	1.02 [0.98-1.05]	1.10 [1.08-1.12]
Employment		
Employed	1.00	1.00
Other/Not Employed	0.94 [0.90-0.96]	0.97 [0.95-0.99]
Marital Status		
Married	1.00	1.00
Other/Not Married	1.18 [1.16-1.22]	1.13 [1.11-1.14]
% Bachelors Education		
Top 50%	1.00 [0.98-1.02]	0.98 [0.96-0.99]
Bottom 50%	1.00	1.00
Median Income		
Top 50%	0.98 [0.98-1.03]	0.97 [0.95-0.99]
Bottom 50%	1.00	1.00

CSM = cancer-specific mortality. OR = odds ratio. SHR = subdistribution hazard ratio. CI = confidence interval. PCP = primary care physician.

© 2022 Qiao E et al. *JAMA Network Open.*

eTable 2. Full Model Outputs of Logistic Regression for Entire Cohort and Annual Primary Care Visits as Endpoint

Characteristic	Odds ratio [95% confidence interval]
Age at Diagnosis	1.05 [1.05-1.05]
Race	
White	1.00
Black	1.12 [1.10-1.15]
Other	0.82 [0.78-0.87]
Ethnicity	
Hispanic	1.09 [1.02-1.16]
Non-Hispanic	1.00
Gender	
Male	1.00
Female	1.50 [1.40-1.61]
Cancer Subtype	
Prostate	1.00
Bladder	1.06 [1.01-1.11]
Breast	1.25 [1.14-1.37]
Colorectal	0.86 [0.84-0.89]
Esophageal	1.12 [1.06-1.18]
Gastric	1.20 [1.11-1.28]
Head and Neck	1.06 [1.02-1.09]
Kidney	1.30 [1.25-1.34]
Liver	1.36 [1.31-1.41]
Lung	1.19 [1.16-1.21]
Melanoma	1.11 [1.06-1.16]
Pancreas	1.25 [1.19-1.31]
Year of Diagnosis	
2004-2008	1.00
2009-2012	1.19 [1.17-1.21]
2013-2017	1.40 [1.38-1.44]
Charlson Score	
0	1.00
1	1.42 [1.38-1.46]
2+	1.48 [1.45-1.51]
Employment	
Employed	1.00
Other/Not Employed	1.18 [1.14-1.20]
Marital Status	
Married	1.00
Other/Not Married	0.90 [0.88-0.91]
% Bachelors Education	
Top 50%	1.02 [1.00-1.04]

© 2022 Qiao E et al. *JAMA Network Open.*

Bottom 50%	1.00
Median Income<br>Top 50%<br>Bottom 50%	 0.94 [0.92-0.95]<br>1.00

© 2022 Qiao E et al. *JAMA Network Open.*

eTable 3. ORs and 95% CIs for Multivariable Logistic Regressions With Metastatic Disease at Diagnosis.

Subtype	OR_{some}, 95% [CI]	*P*	OR_{annual}, 95% [CI]	*P*
Prostate	0.43 [0.40-0.47]	<0.001	0.32 [0.30-0.35]	<0.001
Lung	0.86 [0.81-0.93]	<0.001	0.75 [0.70-0.80]	<0.001
Melanoma	0.43 [0.35-0.53]	<0.001	0.36 [0.29-0.45]	<0.001
Colorectal	0.89 [0.82-0.97]	0.012	0.79 [0.72-0.87]	<0.001
Bladder	0.65 [0.52-0.81]	<0.001	0.55 [0.43-0.69]	<0.001
Gastric	0.77 [0.60-0.98]	0.031	0.64 [0.50-0.82]	<0.001
Kidney	0.76 [0.64-0.89]	<0.001	0.61 [0.52-0.73]	<0.001
Esophagus	0.85 [0.72-1.00]	0.055	0.69 [0.58-0.83]	<0.001
Head and Neck	0.83 [0.69-1.01]	0.061	0.63 [0.51-0.77]	<0.001
Pancreas	1.00 [0.84-1.20]	0.974	0.87 [0.73-1.04]	0.121
Liver	0.68 [0.75-1.06]	0.243	0.70 [0.58-0.84]	<0.001
Breast	0.78 [0.44-1.06]	0.088	0.55 [0.35-0.86]	0.009

Each odds ratio (OR) represents separate multivariable logistic regression for each distinct tumor subtype.

© 2022 Qiao E et al. *JAMA Network Open.*

eTable 4. Comparison of Included vs. Excluded Patients

Variable	Included (n=245,425)	Excluded (n=279,881)
Age, mean (SD), years	65.8 (9.3)	68.0 (9.8)
Gender		
Male	239,560 (97.6%)	274,208 (98.0%)
Female	5,865 (2.4%)	5,673 (2.0%)
Race		
Black	51,468 (22.0%)	55,766 (19.9%)
White	186,716 (76.1%)	215,173 (76.9%)
Other Race[a]	7,241 (2.9%)	8,942 (3.2%)
Ethnicity		
Hispanic or Other[b]	3,938 (1.6%)	3,642 (1.3%)
Non-Hispanic	241,476 (98.4%)	276,239 (98.4%)
Year Diagnosed		
2004-2008	95,101 (38.8%)	67,619 (39.8%)
2009-2012	76,045 (31.0%)	62,311 (36.6%)
2013-2017	74,188 (30.2%)	40,148 (23.6%)
Cancer Subtype		
Prostate	93,456 (38.1%)	102,716 (36.7%)
Lung	48,102 (19.6%)	58,435 (20.9%)
Melanoma	9,612 (3.9%)	11,477 (4.1%)
Colorectal	23,819 (9.7%)	31,345 (11.2%)
Bladder	9,626 (3.9%)	13,306 (4.8%)
Gastric	3,471 (1.4%)	5,471 (2.0%)
Kidney	12,348 (5.0%)	12,033 (4.3%)
Esophagus	6,037 (2.5%)	7,582 (2.7%)
Head and Neck	17,559 (7.2%)	18,591 (6.6%)
Pancreas	6,530 (2.7%)	6,603 (2.4%)
Liver	11,526 (4.7%)	9,069 (3.2%)
Breast	3,339 (1.4%)	3,253 (1.2%)
Employment		
Employed	45,746 (18.7%)	37,556 (13.4%)
Not Employed	199,455 (81.3%)	242,325 (86.6%)
Marital Status		
Married	111,842 (45.6%)	139,273 (49.8%)
Single/Other	133,570 (54.4%)	140,608 (50.2%)
Income, mean (SD), per $1,000	50.1 (18.8)	49.5 (19.1)
Education, mean (SD), college degree % per zip code	15.6 (7.7)	15.2 (7.6)

Excluded patients were limited to those with complete, non-missing demographic data as below.

[a]Race other category includes American Indian, Aleutian, Eskimo, Chinese, Japanese, Filipino, Hawaiian, Korean, Vietnamese, Laotian, Hmong ,Cambodian, Thai, Asian Indian, Pakistani, Micronesian, Chamorro, Guamanian, Polynesian, Tahitian, Samoan, Tongan, Melanesian, Fiji Islander, New Guinean, Other Asian, Pacific Islander, not listed, and unknown

[b]Ethnicity other category includes unknown, not listed, or Spanish surname only

© 2022 Qiao E et al. *JAMA Network Open.*
